# Fusion of purple membranes triggered by immobilization on carbon nanomembranes

**DOI:** 10.3762/bjnano.12.8

**Published:** 2021-01-22

**Authors:** René Riedel, Natalie Frese, Fang Yang, Martin Wortmann, Raphael Dalpke, Daniel Rhinow, Norbert Hampp, Armin Gölzhäuser

**Affiliations:** 1 Faculty of Chemistry and Materials Sciences Center, University of Marburg, Hans-Meerwein-Strasse, D-35032 Marburg, Germany; 2 Physics of Supramolecular Systems and Surfaces, Faculty of Physics, Bielefeld University, Universitätsstraße 25, D-33615 Bielefeld, Germany; 3 Nano Biomaterials Group, Ningbo Institute of Industrial Technology, Chinese Academy of Science, China; 4 Faculty of Engineering and Mathematics, Bielefeld University of Applied Sciences, Interaktion 1, D-33619 Bielefeld, Germany; 5 Max Planck Institute of Biophysics, Department of Structural Biology, Max-von-Laue-Str. 3, D-60438 Frankfurt, Germany

**Keywords:** bacteriorhodopsin, carbon nanomembrane, electrophoretic sedimentation, proton pump, purple membrane

## Abstract

A freestanding ultrathin hybrid membrane was synthesized comprising two functional layers, that is, first, a carbon nanomembrane (CNM) produced by electron irradiation-induced cross-linking of a self-assembled monolayer (SAM) of 4′-nitro-1,1′-biphenyl-4-thiol (NBPT) and second, purple membrane (PM) containing genetically modified bacteriorhodopsin (BR) carrying a C-terminal His-tag. The NBPT-CNM was further modified to carry nitrilotriacetic acid (NTA) terminal groups for the interaction with the His-tagged PMs forming a quasi-monolayer of His-tagged PM on top of the CNM-NTA. The formation of the Ni-NTA/His-tag complex leads to the unidirectional orientation of PM on the CNM substrate. Electrophoretic sedimentation was employed to optimize the surface coverage and to close gaps between the PM patches. This procedure for the immobilization of oriented dense PM facilitates the spontaneous fusion of individual PM patches, forming larger membrane areas. This is, to our knowledge, the very first procedure described to induce the oriented fusion of PM on a solid support. The resulting hybrid membrane has a potential application as a light-driven two-dimensional proton-pumping membrane, for instance, for light-driven seawater desalination as envisioned soon after the discovery of PM.

## Introduction

The so-called purple membrane (PM) has attracted large attention in the scientific literature after this light-driven proton pump was first discovered in the 1970s [1–2]. The membrane patches of about 1 µm in diameter, consisting of the protein bacteriorhodopsin (BR) and lipid molecules [3], can be separated from the cytoplasmic membrane of *Halobacterium salinarum* and have been intensively studied [4–6]. Due to its robustness, PM quickly became one of the best-characterized natural membranes [7]. It is a two-dimensional crystal with a thickness of about 5 nm [8–10]. PM patches are highly resistant towards photochemical and thermal degradation and withstand highly concentrated salt solutions [11–12]. BR consists of seven transmembrane α-helices containing a retinal molecule that is bound to the protein [13–14]. Induced by the light-driven isomerization of retinal, protons are pumped from the cytoplasmic side to the extracellular side in a multi-step photoreaction cycle serving as an energy source for *H. salinarum* [2,15–16].

BR and PM have been extensively studied for technical applications in recent decades [17–22]. However, the fact that PM consists of micrometer-sized patches only represents a significant limitation of potential applications. Another limitation of the technical usability is the need for oriented alignment of the patches. Due to the vectorial transport of protons through PM, the unidirectional orientation of the patches is an important requirement for a functional device [8]. Although various methods have already been tested with varying degrees of success in terms of orientation [23–28], the fusion of oriented membrane patches into a single, free-standing, large-area PM monolayer remains challenging. Previous approaches for the oriented assembly of PM were based on solid supports [8,29–30] or interfaces between liquids [31–32]. Other approaches, by implementing transmembrane proteins such as BR in lipid bilayers, have so far been used only to study protein behavior and also do not provide the means to construct an oriented PM monolayer for potential technical applications [33–34].

In this work, we report the successful assembly of a unidirectionally oriented PM quasi-monolayer on an ultrathin carbon nanomembrane (CNM) substrate. CNMs are stable freestanding monolayers produced from irradiation-induced cross-linking of self-assembled monolayers (SAMs) [35]. They have a thickness in the range of 1–2 nm and can be made from a variety of carbon precursor molecules [36]. They are well characterized in terms of their permeability [37–38] and optical properties as well as their chemical [39], thermal [40], and mechanical stability [41]. PM patches were deposited by electrophoretic sedimentation from a suspension as a quasi-monolayer on a 4′-nitro-1,1′-biphenyl-4-thiol (NBPT) CNM [42–43]. We use the term quasi-monolayer to express that there is essentially one monolayer of PM patches on the CNM, but the coverage is less than 100% and in some regions irregularly arranged PM patches overlap. Immobilization of PM was achieved by complex formation of nickel(II) nitrilotriacetic acid (Ni-NTA), coupled to the NBPT CNM, with the C-terminal histidine-tag at the extracellular side of a PM mutant (c-His PM). The functionalization and the resulting hybrid membrane were examined by atomic force microscopy (AFM), scanning electron microscopy (SEM), X-ray photoelectron spectroscopy (XPS), confocal laser scanning microscopy (CLSM), and infrared reflection absorption spectroscopy (IRRAS).

## Experimental

### Preparation of SAMs and CNMs

NBPT was purchased from Taros Chemicals (Dortmund, Germany). Thermally evaporated Au films (300 nm) on mica supports (Georg Albert physical vapor deposition coatings) were used as substrates for the SAM preparation. Cleaning of the Au surfaces was achieved with an UV/ozone-cleaner (FHR Anlagenbau GmbH, UVOH 150 LAB) and subsequent immersing in absolute ethanol (AnalaR NORMAPUR, VWR Chemicals). The SAMs were prepared by immersion of the cleaned Au substrate into a solution (approx. 10 mM) of 4′-nitro-1,1′-biphenyl-4-thiol in dried and degassed *N*,*N*-dimethylformamide (DMF, anhydrous, 99.8%, Sigma-Aldrich) under nitrogen atmosphere in a sealed flask for 72 h, followed by subsequent rinsing with DMF (BioSyn, ≥99.9%, Honeywell) and absolute ethanol. Cross-linking of the SAMs was conducted by exposing the SAM to 100 eV electrons (SPECS flood gun) in high vacuum (10^−7^ mbar) using doses of 50 mC/cm^2^. The cross-linking of a NBPT SAM on gold on mica leads to an NBPT CNM.

### Preparation of PM

Frozen wild-type (WT) and c-His-tagged purple membrane were thawed, vortexed and subsequently diluted to the desired optical density (OD) using distilled water. The OD was measured at 589 nm using a UV–vis spectrometer (Lambda 35, Perkin Elmer).

### Sample preparation

Drop casting: PM solutions of different volumes and concentrations were dripped onto the CNM substrates and incubated for different periods. Drying was prevented by putting the substrate into a closed container along with a small vessel filled with distilled water. After incubation, the substrate was gently rinsed with 200 µL distilled water and then dried in a mild nitrogen stream.

Electric field sedimentation: Electric field sedimentation experiments were done using a capacitor consisting of the CNM substrate as the upper and an indium tin oxide (ITO) as the lower capacitor plate. A sample drop was placed between the two electrodes. For electrostatic experiments (drop was in contact with substrate plate only) the plate separation distance was held constant at 10 mm, while it was reduced to 2 mm for electrophoretic experiments (drop was in contact with both plates). Since WT PM is negatively charged on both sides, the substrate plate was chosen as the positive pole of the capacitor to attract the PM sheets. To enhance binding the positively charged side of c-His PM to the Ni-NTA-complex of the substrate, the substrate plate was chosen as the negative pole.

After the preparation of the PM solution, the capacitor was charged using a standard lab power supply and a drop of PM solution was injected either onto the CNM substrate or in between both plates. After a certain time (incubation time), the drop was removed using a syringe. The voltage was held constant for another period (drop post-removal time, DPR time) to counteract drying effects. Before analysis, the substrate was gently rinsed with 200 µL distilled water and then dried in a mild nitrogen stream. Experiments were carried out with different drop sizes, PM optical densities, incubation times, DPR times, and applied voltages.

### CNM functionalization

The CNM was placed into a dried flask with 1 mL 2-azidoacetyl chloride (2-AAC, 97%, 30% solution in diethyl ether, SelectLab Chemicals GmbH, Münster, Germany), 8 mL dichloromethane (CH_2_Cl_2_, anhydrous, ≥99.8%, 50–150 ppm amylene, Sigma-Aldrich, Taufkirchen), and 0.3 mL diisopropylethylamine (DIPEA, ≥99%, Sigma-Aldrich). The flask was cooled in an ice bath to slow down the reaction and to dissipate the heat of the reaction. After 24 h the reaction has been quenched with absolute ethanol and the sample was rinsed with ethanol and isopropanol (AnalaR NORMAPUR, VWR Chemicals, Darmstadt, Germany). Afterwards, the sample was immersed in 1 mg of dibenzocyclooctyne-maleimide (DBCOM, >88%, Sigma-Aldrich) in 20 mL dried DMF solution. After 24 h of incubation protected from light, the sample was rinsed with ethanol and dried in a stream of nitrogen. The sample was further functionalized by immersion in 1 mg of *N*-[*N*,*N*-bis(carboxymethyl)-ʟ-lysine]-12-mercaptododecan-amide (≥90%, Sigma-Aldrich) in 15 mL dried DMF solution. The sample was stored protected from light for 24 h. Afterwards the sample was rinsed with ethanol, dried in a stream of nitrogen, and stored under argon gas.

### Sample characterization

X-ray photoelectron spectroscopy (XPS) has been conducted in an Omicron Multiprobe UHV system (Scienta Omicron GmbH, Taunusstein, Germany) using monochromatic Al Kα irradiation, a Sphera electron analyzer with a resolution of 0.9 eV and an emission angle of 20°. AFM measurements were performed with a Bruker NanoScope^®^ V system (Bruker Corporation, Billerica, MA, USA) in PeakForce mode using a PicoForce scanner for 50 × 50 µm^2^ topographical pictures. A Multimode 8 10364EVLR scanner was used for topographical pictures with a smaller field of view as well as for electrostatic measurements. For PeakForce Kelvin probe force microscopy (PF-KPFM), a line is first scanned in PeakForce mode. In a second scan the same line is scanned with an adjustable height offset and using the topography data from the first scan. The voltage applied to the tip to compensate for attracting/repelling electrostatic forces indicates the electrostatic potential of the sample.

The samples were further analyzed by SEM in a Zeiss Auriga (Carl Zeiss, Jena, Germany) at an acceleration voltage of 5 kV using the in-lens detector for secondary electrons. For the visualization of fluorochromes a confocal laser scanning microscope CLSM 780 (Carl Zeiss, ibid.) has been used. The IRRAS spectrum was recorded using a nitrogen-purged Bruker Vertex70 spectrometer (Bruker Corporation, ibid.) with a PMA50 polarization modulator unit. Further SEM images were recorded using a Vega W-REM from Tescan. UV–vis data was further processed and evaluated using NanoScope Analysis 1.90 (Build R1.135067, Bruker Corporation, ibid.), ImageJ 1.51j8 (National Institutes of Health, USA) and Gwyddion 2.47. Graphs were plotted using OriginPro 8 SR0 v8.0724 (OriginLab Corporation, Wellesley Hills, MA, USA).

## Results and Discussion

### Improving substrate coverage and PM orientation

An oriented deposition of the PM patches is required to take advantage of the unidirectional proton transferability of PM. Preliminary experiments were performed to obtain both optimal orientations of the PM patches and coverage of the CNM substrate. Due to the limited availability of c-His PM, wild-type PM (WT PM) has been used for all preliminary experiments. It has been found that the most appropriate CNM precursor for this purpose is NBPT since the synthesis route is well established and the resulting CNM has reactive amino head groups that can be functionalized in subsequent experiments. The functionalization of the CNM is necessary to immobilize the PM patches by chemical complex formation after deposition.

**Figure 1:**
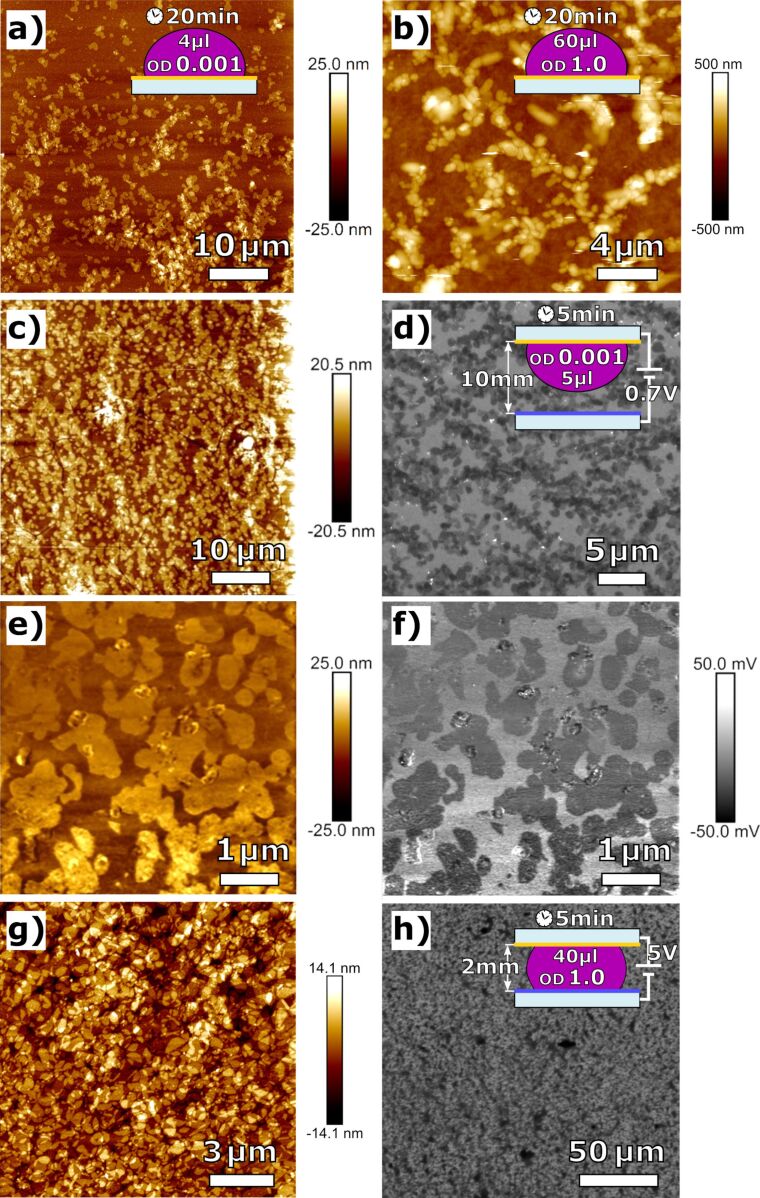
WT PM deposited on NBPT CNM using drop-casting and electrophoretic sedimentation: (a, b) initial drop-casting experiments showing a low substrate coverage of approx. 35% and/or cluster formation. (c–f) Sedimentation in an electric field: (c, e) AFM image at different magnifications, (d) SEM image and (f) PF-KPFM image showing the uniform orientation of the PM fragments with approx. 60% substrate coverage. (g, h) AFM and SEM images of a WT PM quasi-monolayer deposited on NBPT using electrophoretic sedimentation resulting in 98% substrate coverage. A height profile of (g) can be seen in Supporting Information File 1.

In a first step, simple drop-casting experiments, as illustrated in Figure 1a,b with WT PM have been performed to optimize the substrate for a high coverage ratio. For this, the CNM substrates were prepared by deposition of 60 µL WT PM suspension in distilled water with different optical densities (OD) for 20 min on the NBPT CNM substrate. The samples were subsequently washed with 200 µL distilled water and dried in a nitrogen flow. For each sample the topography of a 50 × 50 µm^2^ area was investigated by AFM to evaluate substrate coverage. It has been shown that the WT PM patches do not form a continuous monolayer, but rather separately distributed patch islands at low OD, or large patch clusters at high OD. The drop has been preserved in a small container along with a small vessel filled with water to prevent drying. A coverage rate of more than 90% could be achieved, but AFM imaging has shown that a higher coverage rate is associated with a higher rate of multilayers and PM clusters. To decrease the number of clusters, both OD and drop size have been reduced as larger drops lead to higher coverage rates due to increasing amounts of WT PM per unit area.

In order to improve the deposition and to gain well-oriented WT PM, as shown in Figure 1c–f, an electrical field was applied to the drop. The substrate itself served as a positively charged capacitor plate whereas the negatively charged plate consisted of indium tin oxide. As the electric field is attenuated in areas that have already been covered by PM patches, subsequently deposited patches primarily prefer uncovered areas. In addition, the PM gets well oriented in an electric field according to its surface charge. Initially, a drop of 5 µL WT PM suspension was attached to the substrate only and was not in contact with the opposite electrode. While the plate distance was kept constant at 10 mm, OD and voltage were varied to increase substrate coverage and orientation. As seen in Figure 1c–f, complete orientation with improvable coverage has been achieved.

To prevent cluster formation of the WT PM patches at higher OD, the distance between the capacitor plates was reduced to 2 mm in a subsequent step so that the drop was in contact with both capacitor plates and a current could flow through the WT PM suspension as shown in Figure 1h. The WT PM was thus electrophoretically deposited onto the NBPT CNM substrate. 40 µL of a WT PM suspension with an OD of 1.0 was inserted between the capacitor plates using a syringe. For an incubation time of 5 min, a voltage of 5 V was applied, and then the drop was removed. Since the substrate was not completely dry after removal of the solvent, it was found that the surface tension pulls the deposited patches apart forming quasi-periodic concentric displacements of PM patches, see Figure S1, Supporting Information File 1. To counteract those drying effects, the electric field was maintained for additional 5 min DPR time. As can be seen in Figure 1g,h, a homogeneously distributed monolayer of uniformly oriented WT PM patches with only a few vacancies could be obtained.

### Assembly of a stable hybrid membrane

**Figure 2:**
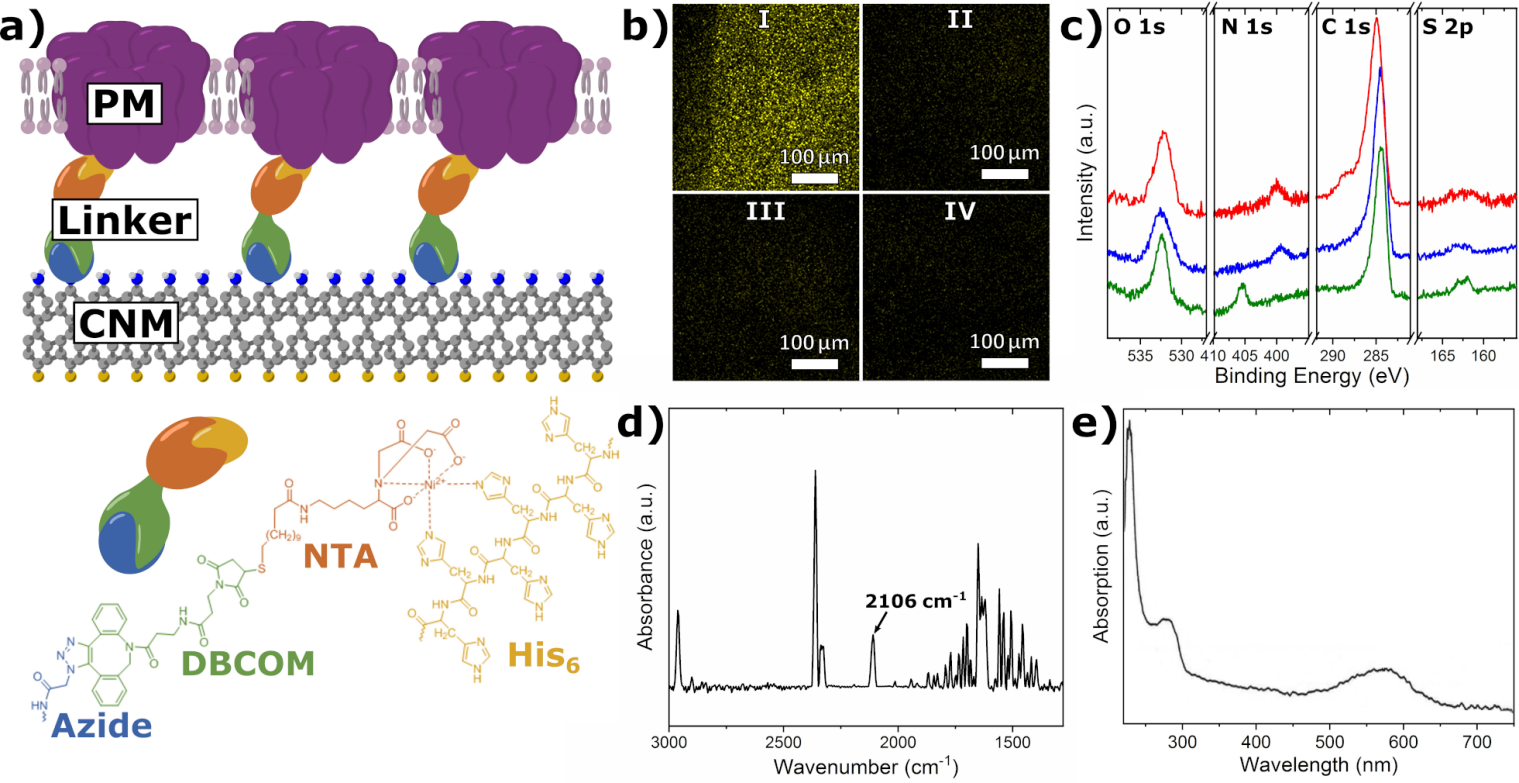
(a) Illustration of a hybrid structure consisting of an NTA CNM and a c-His PM. The c-His PM consists of BR (purple) with a lipid bilayer between the BR molecules. The chemical structure shows the linker used to build a complex between NTA CNM and c-His PM. (b) Fluorescence microscopy of NTA-functionalized NBPT CNMs incubated with a fluorescent protein (mTorqoise): I. Functionalized NBPT CNM sample loaded with nickel and incubated with the protein. The edge of the drop can be seen on the left side. II. The same sample after elution with imidazole. III. Without nickel, incubated with the protein. IV. The same sample after elution with imidazole. A strong fluorescence signal in I. indicates that the functionalization was successful. (c) XPS spectra of the NBPT SAM (green), NBPT CNM (blue), and NBPT CNM after the NTA functionalization (red). (d) IRRAS spectrum of NBPT CNM after the first functionalization step. (e) UV–vis absorption spectrum of a c-His PM solution. The absorption peak at 570 nm indicates that BR is functional.

The experiments were repeated with a genetically modified species of c-His PM. This species has histidine-tags on every BR molecule and hence is strongly positively charged on the extracellular side. Thus, a chemical complex bond formation between NBPT and c-His PM can be obtained. The histidine-tag binds to the substrate via a Ni-NTA linkage as schematically shown in Figure 2a. The NBPT CNM substrates were functionalized to obtain NTA head groups. The functionalization route is explained in the Experimental section and the final linker is shown in Figure 2a.

The functionalization was tested with the His-tagged fluorescent protein mTurqoise. Confirmation of successful functionalization was provided by fluorescence imaging as seen in Figure 2b. The images show that the complex bonds formed with His-labeled proteins are reversible. Furthermore, the functionalization was investigated utilizing XPS and IRRAS as seen in Figure 2c,d. The IRRAS spectrum of an NBPT CNM after the first functionalization step reveals a peak at 2106 cm^−1^ caused by the asymmetrical stretching vibration of the azide moiety, which indicates that the first functionalization step was successful. Figure 2 shows the XPS spectra of the pristine NBPT SAM (green), the cross-linked NBPT CNM (blue), and the NTA-functionalized NBPT CNM (red). The conversion of a SAM to a CNM can be traced by the chemical shift of the N 1s binding energy from 405.5 to 399.2 eV, by the broadening of the oxygen and carbon peak, and by additional sulfur species belonging to thiol groups no longer bound to gold after cross-linking. The presence of oxygen in the CNM spectrum is due to atmospheric contamination and can theoretically be avoided by preparation in high vacuum [36]. The functionalization (red) is indicated by a carbon shoulder around 289 eV referring to carboxyl groups of the NTA moieties [44]. The increasing signal intensities of the carbon and oxygen peaks indicate a successful functionalization. The nitrogen peak shows a constant intensity accompanied by a higher secondary electron background. This is a direct result of an increasing layer thickness due to the functionalization, as the functionalized layer contains fewer nitrogen atoms per volume than the CNM.

For electrophoretic sedimentation experiments, the same conditions as for WT PM (Figure 1h) were used. Unlike the WT PM, c-His PM tends to form clusters rather than a monolayer. This might be explained by the fact that the c-His PM has oppositely charged sides, which makes them attract and adsorb to each other [28]. Attempts to solve this problem by using a buffer solution to shield the surface charges have shown that homogeneous monolayer formation is impeded by buffer crystal formation.

Instead, sedimentation of c-His PM has been performed with a much more dilute suspension of OD 0.02. The incubation time was reduced to 3 min and the voltage was held constant at 5 V. To maintain high substrate coverage despite low OD, the incubation was repeated four times.

**Figure 3:**
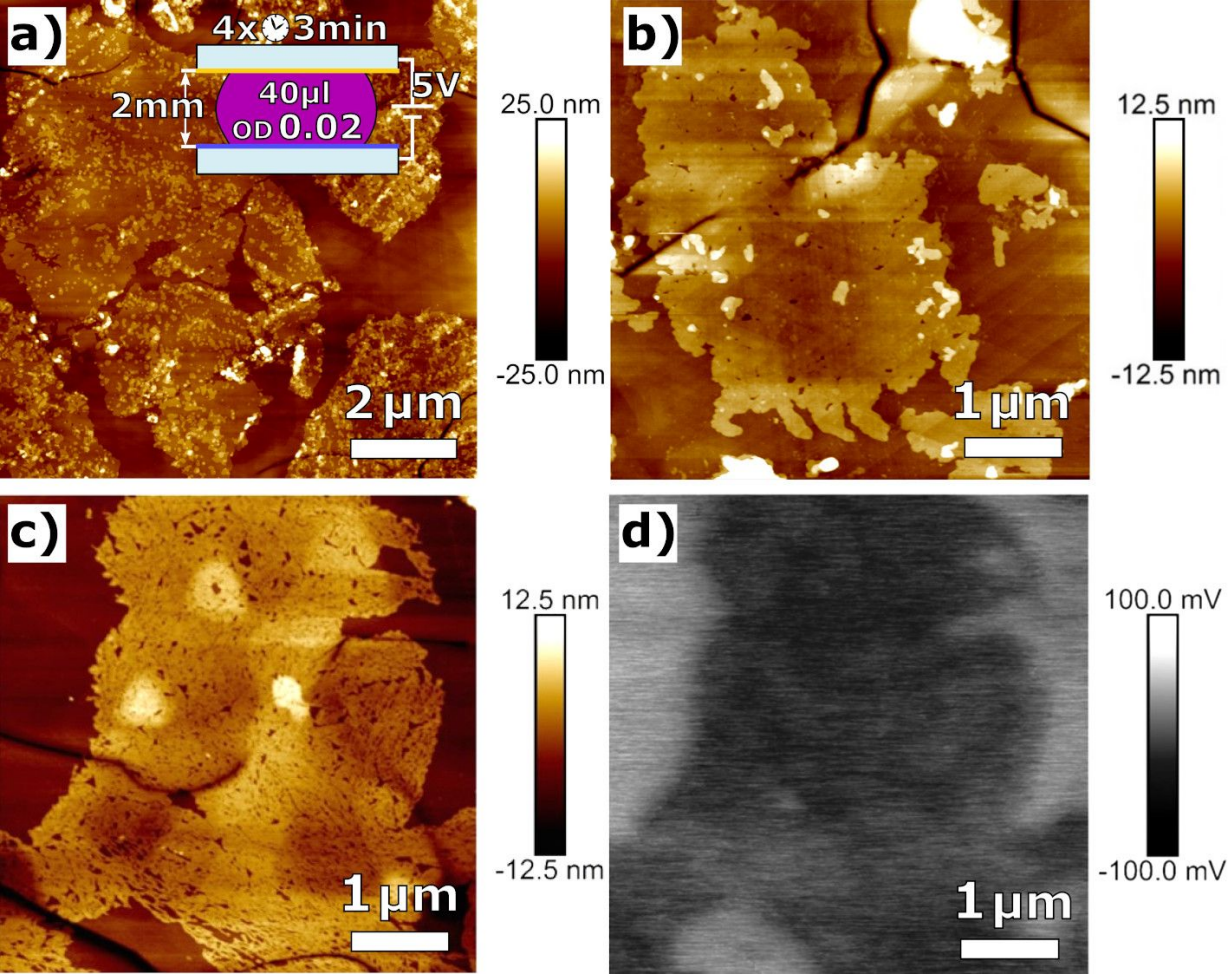
(a–c) Large-area agglomerates of c-His PM forming an immobilized quasi-monolayer with only a few vacancies on a functionalized NBPT CNM. (d) The electrostatic potential of the area shown in (c) reveals a uniform orientation of c-His PM.

Despite the reduction of optical density, large areas of merged c-His PM monolayer could be observed as shown in Figure 3. The fused PM sheets showed small vacancies and fissures, however, the constant height of 5.0–5.2 nm indicated an intact crystalline structure. As expected from preliminary experiments, the measurements of the electrostatic potential shown in Figure 3d revealed a uniform orientation of PM.

To further explore the fusion mechanism that led to the formation of a PM quasi-monolayer, the effects of different parameters have been investigated. Fusion first needs uniform orientation. This is accomplished by the His-tag. The lipid composition of the outer and the inner side of the PM differs. In the second step, the recrystallization of the PM patches starts in areas where they were overlapping after deposition. Some parts of the PM sheet laying on top are lost. In addition, OD, voltage, incubation time, and DPR time were varied, while plate distance and drop size were held constant at 2 mm and 40 µL, respectively.

At 5 V and OD 0.02, the ideal incubation time was found to be between 3 and 4 min. Samples that were prepared with incubation time less than 3 min did not show any fusion. Instead, cluster formation and bending of single PM sheets were observed. An incubation time of more than 4 min results in the decomposition of the fused regions. After 5 min or more small pieces of circularly ordered PM fragments occur. Here, bubbles may have been formed through electrolysis.

It has been found that the fusion mechanism also depends on the DPR time. After 3 min of DPR time first connections between the different PM patches can be observed indicating incipient fusion. No PM fusion could be observed when the DPR time was omitted. Instead, big piles of single PM sheets appeared due to drying effects. This shows that the DPR time seems to play a critical role in the fusion process. After a high DPR time fusion seemed to take place, but also clusters were formed. A monolayer of lipids with a height of about 2.5 nm (which is about the height of a PM lipid monolayer) is left on the substrate, which may have been a fully monolayered c-His PM before. The optimum DPR time is about 4 min.

An investigation of the effects of applied voltage on the fusion phenomenon has shown that at low voltages of up to 0.5 V no fusion but rather agglomeration is observed. Above 10 V, the patches seem to decay into smaller pieces, see Figure S4, Supporting Information File 1. A voltage of 5 V is optimal regarding the fusion results as shown in Figure 3.

OD had to be low to prevent cluster formation. The number of merged areas decreased with reduced OD. This can be explained by the fact that PM sheets must get into contact with each other to fuse. Hence, the PM concentration does not influence the fusion mechanism itself.

From the observations, a possible c-His PM fusion formation procedure can be assumed. At the initial moment of drop injection of the PM solution to the capacitor, the PM sheets are homogenously distributed. The sheets are bent due to the strong positive charges on one side of the sheets. As the electric field takes effect, the PM sheets adjust to the orientation of the electric field according to their surface charges. PM starts moving towards the substrate. The electric field strength acting on a PM sheet is reduced by formerly adsorbed PM sheets on the substrate, making it more prone to move to an empty substrate area. Depending on the field strength, the bending of the PM sheets is straightened at the substrate. Since no fusion could be observed without DPR time, merging seems to start when the solution drop is removed. PM crystallinity is preserved upon fusion as it is known that noncrystalline PM has a height of 4 nm only [45]. As the fusion process continues, pile stacks build from the upper lipid monolayer of the PM, leaving huge PM monolayer areas on the substrate. Variation of the electric field strength seems to affect the speed of the processes.

## Conclusion

A proof of concept has been demonstrated for the fabrication of a large-area hybrid nanoscale membrane of unidirectionally oriented and, at least partially, fused PM patches on a functionalized NBPT CNM substrate. This opens new possibilities for the technical application of PM. Both, substrate coverage rate and PM orientation, have been investigated depending on the process parameters of electrophoretic sedimentation. An electric field between the substrate and an ITO electrode has been used for the controlled deposition of genetically modified c-His PM from a suspension. The best results were achieved after the incubation of 40 µL c-His PM suspension of OD 0.02 at 5 V for 3 min and a DPR time of 5 min at a capacitor plate distance of 2 mm. Immobilization of the resulting PM quasi-monolayer has been achieved by the complex formation through Ni-NTA between the functionalized CNM and the C-terminal His-tag of PM. Interconnected regions of c-His PM of more than 15 µm in diameter have thus been obtained. We are optimistic that complete substrate coverage can be achieved through further optimization measures. One approach would be to alternate the polarity of the electric field several times after deposition to remove unbound PM patches.

## Supporting Information

Additional AFM images for different deposition parameters and height profiles of AFM images in Figure 1g and Figure 3b.

File 1 Additional figures.
