# Supplementary material for: Fusion of purple membranes triggered by immobilization on carbon nanomembranes
Source: Beilstein J Nanotechnol. 2021 Jan 22;12:93–101. doi: 10.3762/bjnano.12.8 (PMC7849249; doi:10.3762/bjnano.12.8)
Supplement: File 1 — Additional figures. [file Beilstein_J_Nanotechnol-12-93-s001.pdf]

BEILSTEIN JOURNAL OF NANOTECHNOLOGY

Supporting Information

for

Fusion of purple membranes triggered by immobilization on carbon nanomembranes

René Riedel, Natalie Frese, Fang Yang, Martin Wortmann, Raphael Dalpke, Daniel Rhinow, Norbert Hampp and Armin Gölzhäuser

Beilstein J. Nanotechnol. **2021,** *12,* 93–101. doi:10.3762/bjnano.12.8

Additional figures

License and Terms: This is a supporting information file under the terms of the Creative Commons Attribution License (https://creativecommons.org/licenses/by/4.0). Please note that the reuse, redistribution and reproduction in particular requires that the author(s) and source are credited and that individual graphics may be subject to special legal provisions.
The license is subject to the *Beilstein Journal of Nanotechnology* terms and conditions: (https://www.beilstein-journals.org/bjnano/terms)

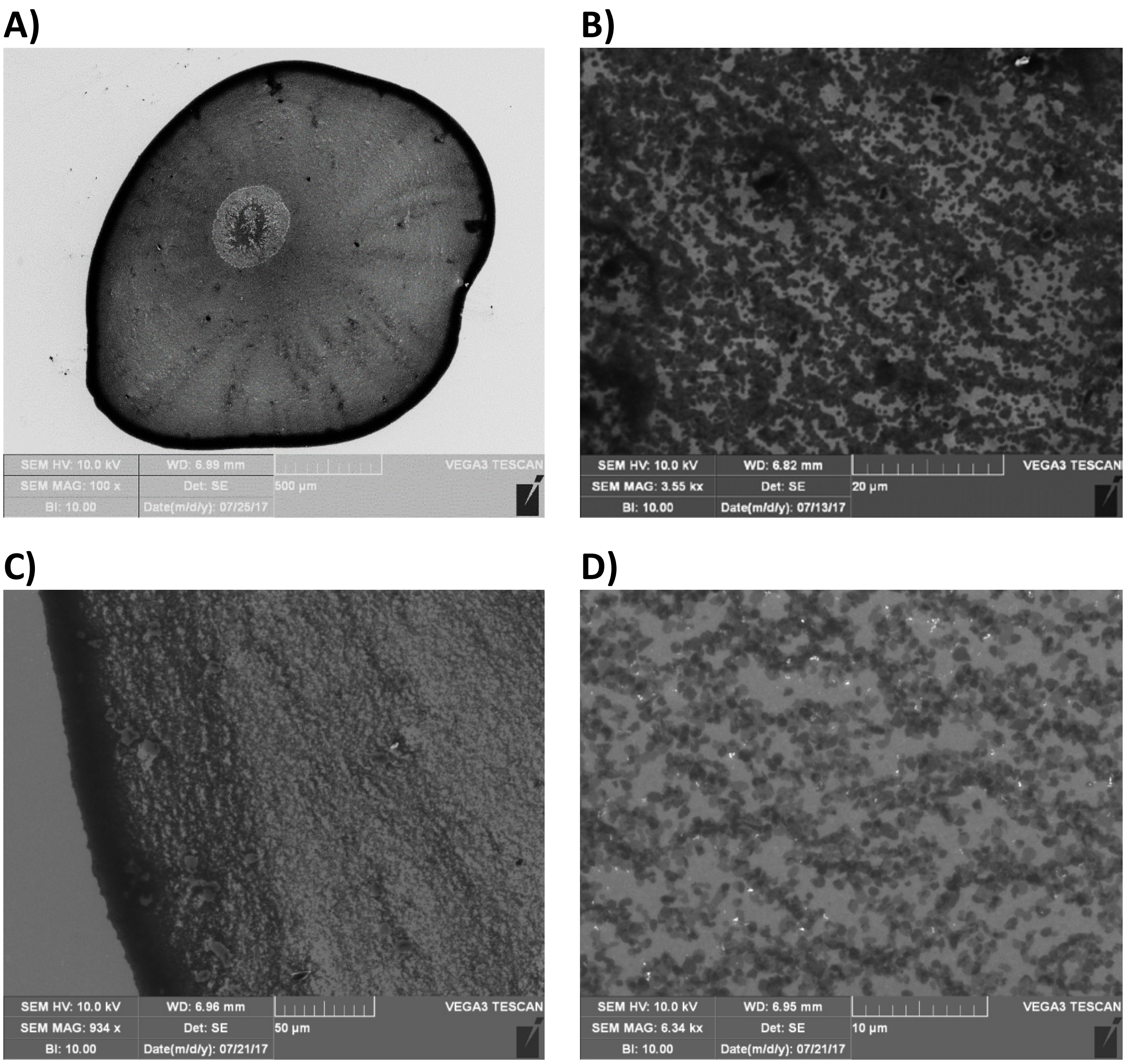

Figure S1: Drying effects become apparent by reorientation of the PM patches. In a shrinking drop, the surface tension pulls the deposited patches apart again if the electric field is turned off. As can be seen in the SEM images below, concentric displacements of PM patches are formed due to drying effects (see B and D). Our experiments have shown that during DPR the electric field is sufficient to attach the patches to the substrate.

The images below show AFM measurements of c-His PM after electrophoretic sedimentation on NiNTA-CNM-substrate using different parameters.

A)

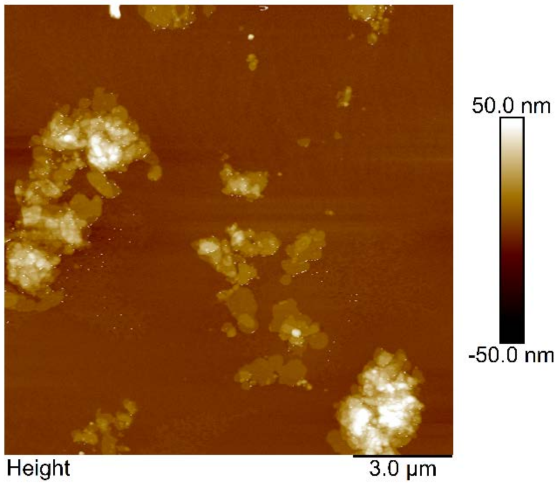

B)

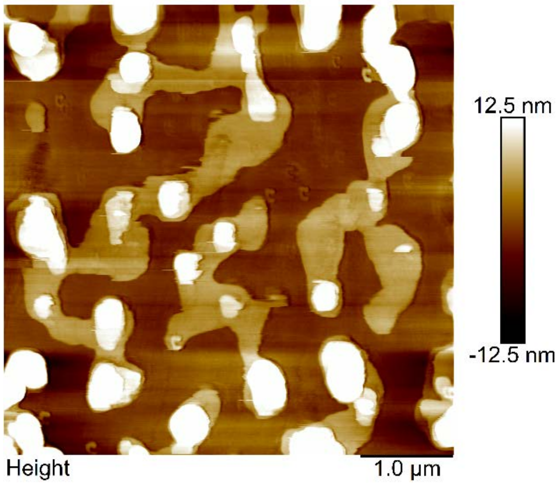

C)

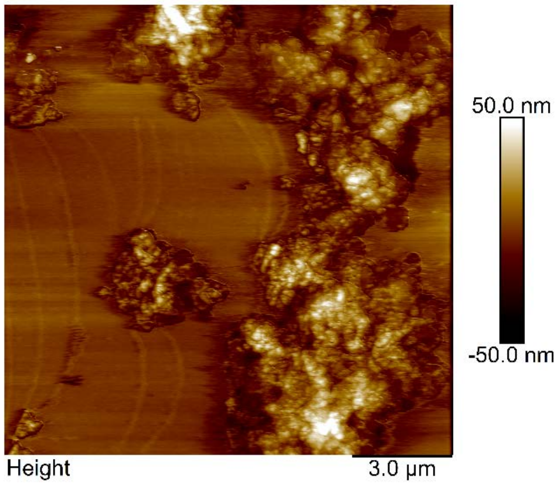

D)

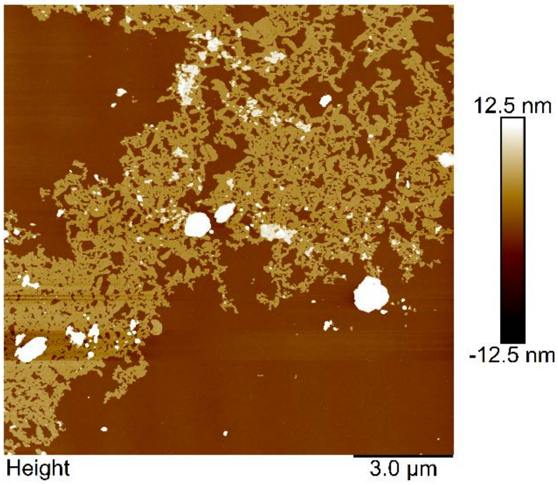

E)

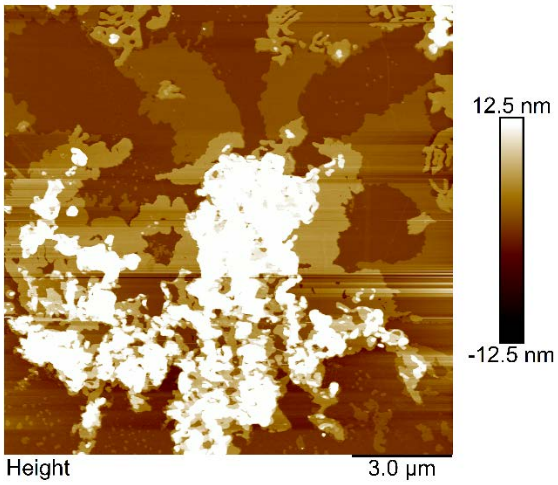

F)

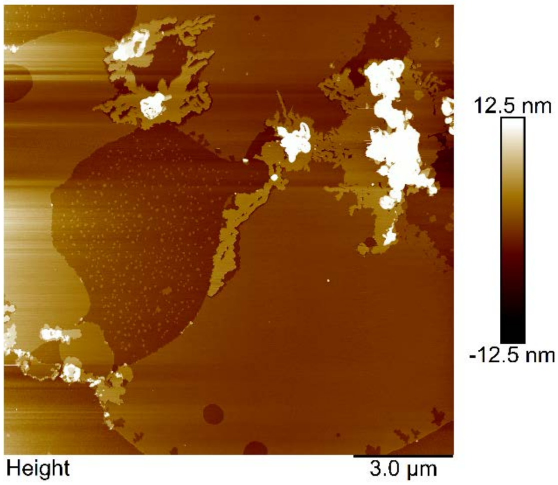

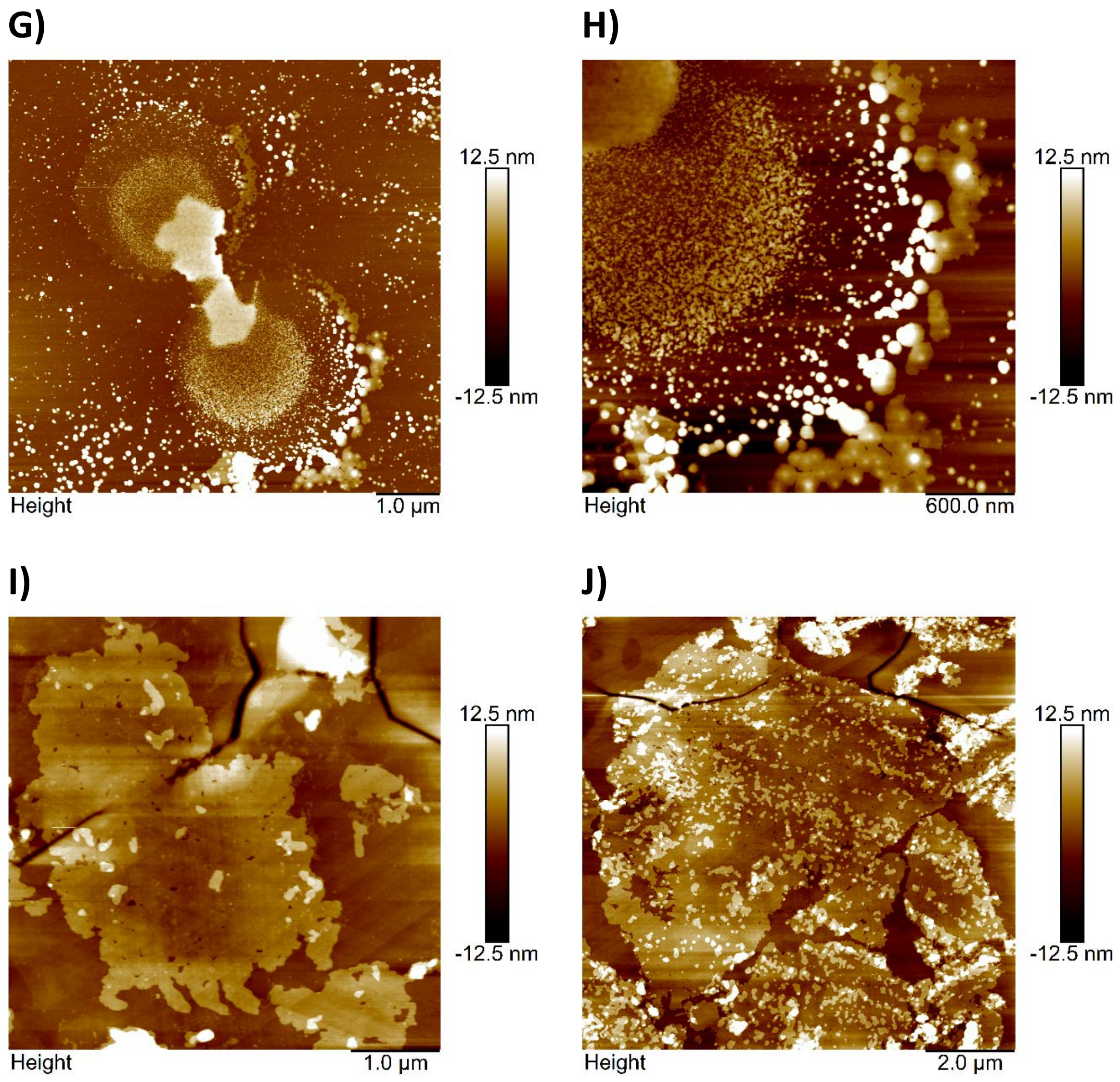

Figure S2: Atomic force microscopic images of the topographies of electrophoretically deposited c-His PM. The capacitor voltage was 5 V, the optical density was 0.02. In the following, the times are given as incubation time + DPR time. A) 1 + 5 min; B) 3 + 3 min; C) 4 + 0 min; D) 4 + 4 min; E) 4 + 8 min; F) 4 + 8 min; G) 5 + 10 min; H) 5 + 10 min; I) 3 + 5 min; J) 3 + 5 min. It can be seen that fusion progressed with increasing incubation time (A, I, G). DPR time was necessary to counteract drying effects, but too much DPR time led to dissolution of the bilipid monolayer with pile formation. Optimal results were obtained after 4 min incubation time and 5 min drying time.

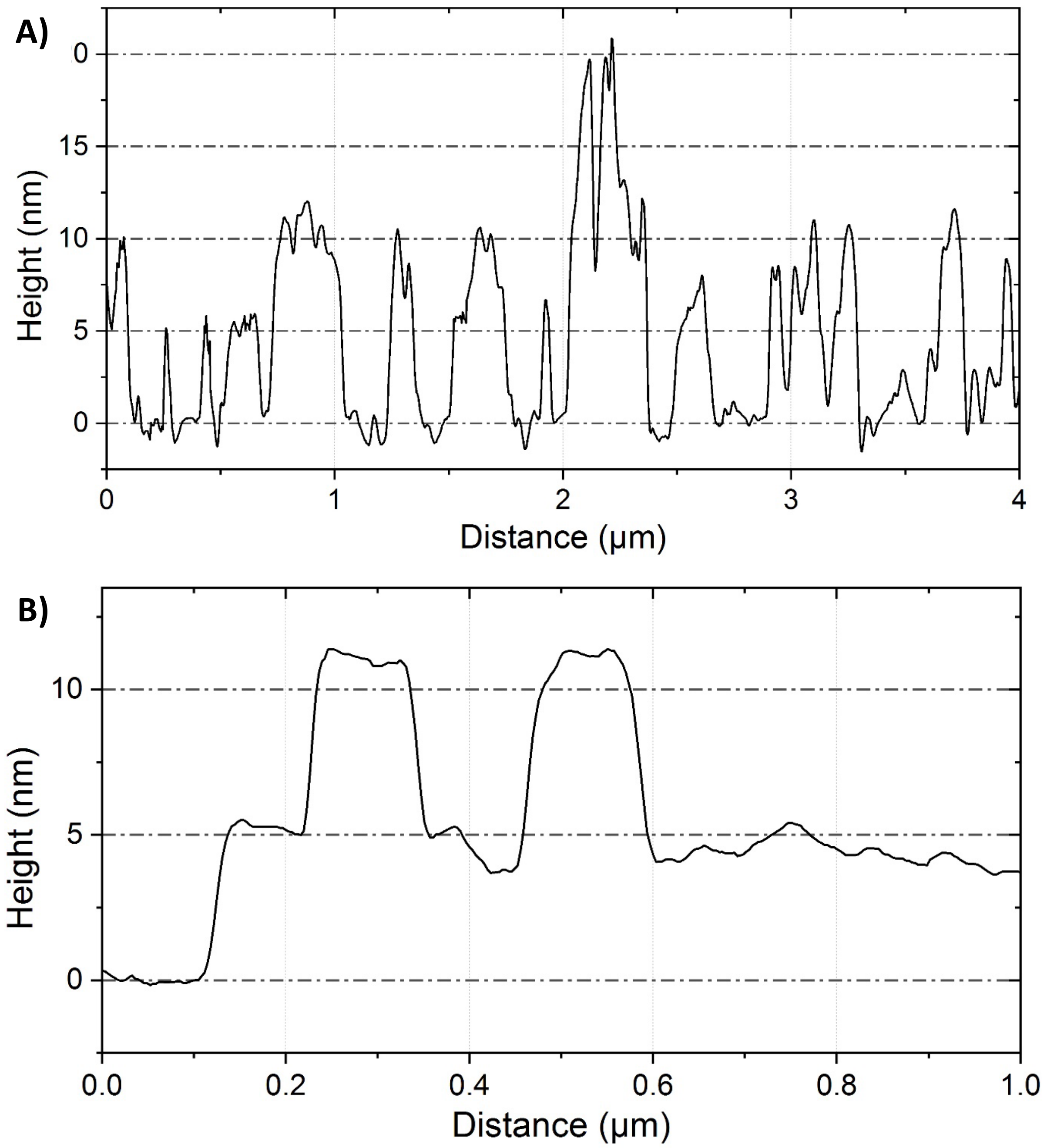

Figure S3: Height profile of A) Figure 1g and B) Figure 3b. The horizontal lines indicate the number of PM layers, corresponding to about 5 nm layer thickness per PM monolayer.

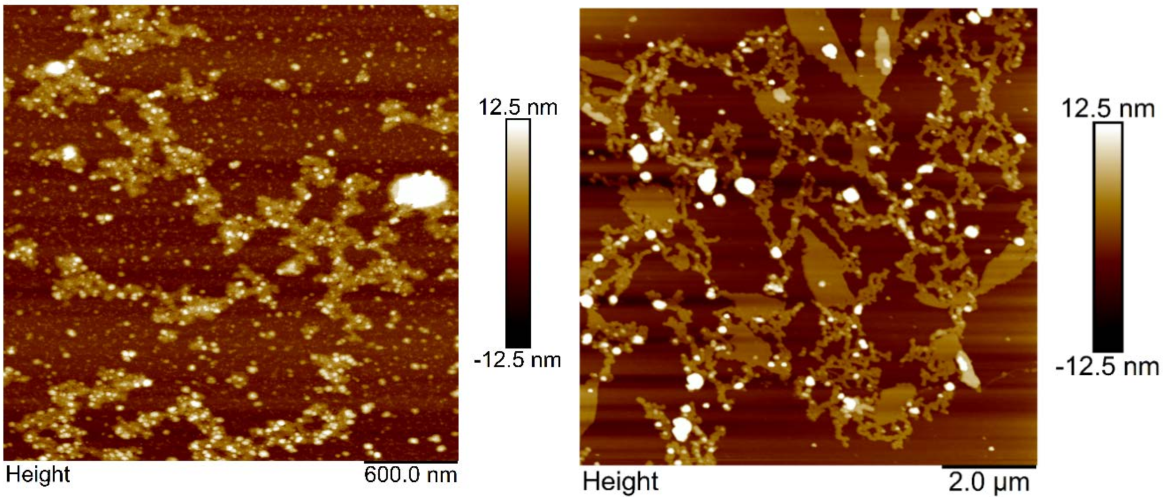

Figure S4: AFM images show the deposited c-His PM at applied voltages of 0.5 V (left) and 10 V (right).
